# Biological Impact of γ-Fe_2_O_3_ Magnetic Nanoparticles Obtained by Laser Target Evaporation: Focus on Magnetic Biosensor Applications

**DOI:** 10.3390/bios12080627

**Published:** 2022-08-11

**Authors:** Fedor A. Fadeyev, Felix A. Blyakhman, Alexander P. Safronov, Grigory Yu. Melnikov, Anastasia D. Nikanorova, Iuliia P. Novoselova, Galina V. Kurlyandskaya

**Affiliations:** 1 Department of Biomedical Physics and Engineering, Ural State Medical University, 620028 Ekaterinburg, Russia; 2 Institute of Medical Cell Technologies, 620026 Ekaterinburg, Russia; 3 Institute of Natural Sciences and Mathematics, Ural Federal University, 620002 Ekaterinburg, Russia; 4 Institute of Electrophysics UB RAS, 620016 Ekaterinburg, Russia; 5 Institute of Human Genetics, Medical Faculty, University Hospital Düsseldorf, Heinrich-Heine-University Düsseldorf, 40225 Düsseldorf, Germany; 6 Departamento de Electricidad y Electrónica, Universidad del País Vasco UPV/EHU, 48080 Bilbao, Spain

**Keywords:** magnetic nanoparticles, constant magnetic field, biomedical applications, human skin fibroblasts, cell proliferation, cytokine secretion

## Abstract

The biological activity of γ-Fe_2_O_3_ magnetic nanoparticles (MNPs), obtained by the laser target evaporation technique, was studied, with a focus on their possible use in biosensor applications. The biological effect of the MNPs was investigated in vitro on the primary cultures of human dermal fibroblasts. The effects of the MNPs contained in culture medium or MNPs already uptaken by cells were evaluated for the cases of the fibroblast’s proliferation and secretion of cytokines and collagen. For the tests related to the contribution of the constant magnetic field to the biological activity of MNPs, a magnetic system for the creation of the external magnetic field (having no commercial analogues) was designed, calibrated, and used. It was adapted to the size of standard 24-well cell culture plates. At low concentrations of MNPs, uptake by fibroblasts had stimulated their proliferation. Extracellular MNPs stimulated the release of pro-inflammatory cytokines (Interleukin-6 (IL-6) and Interleukin-8 (IL-8) or chemokine (C-X-C motif) ligand 8 (CXCL8)) in a concentration-dependent manner. However, the presence of MNPs did not increase the collagen secretion. The exposure to the uniform constant magnetic field (H ≈ 630 or 320 Oe), oriented in the plane of the well, did not cause considerable changes in fibroblasts proliferation and secretion, regardless of presence of MNPs. Statistically significant differences were detected only in the levels of IL-8/CXCL8 release.

## 1. Introduction

Magnetic biosensors, as advanced diagnostic tools, were introduced to the field of biomedical research long ago [1,2,3]. Biomedicine requests three different types of applications: analysis of electric and/or magnetic properties of biological systems focused on their functionality, analysis of the selected properties of the bioanalytes, and analysis of properties of biosystems with magnetic indicators. The last direction is a relatively new, fast-growing field related to the biological objects with complex structures revealed by the specific uptake of magnetic nanoparticles (MNPs). In order to explain this concept, a simple example can be provided. In our previous work [4], electrostatically stabilized water based ferrofluid was prepared using γ-Fe_2_O_3_ magnetic nanoparticles obtained by laser target evaporation and sodium citrate. As a result of the interaction of such a ferrofluid in the concentration of 100 maximum permissive dose with multipotent mesenchymal stromal cells, single inclusions were present in the cellular cytoplasm. In addition, aggregates of the MNPs were noted inside the endosomes, contouring the hydrolytic vesicles and/or outer membrane of the mitochondria and secretory granules. However, large aggregates were accumulated in the mitochondria, which looked similar to myelin bodies. In this case, MNPs anisotropic distribution revealed structural peculiarities of the cell (magnetic indicator case). Sometimes it is difficult to differentiate the type of the detection process. For example, testing the concentration of the MNPs inside living cells involves a large number of cells. The same is true for the MNPs inside ferrogel. Both cases can be considered to be the analysis of the selected properties [5,6]. However, the analysis of the geometry of ferrogel samples situated inside the hydrogel (mimicking the whole tumor geometry detection) is, rather, the magnetic indicator case. There is extended literature related to the analysis of electric and/or magnetic properties of biological systems by biosensors [7,8]. Here, we mention this type of applications just for completeness.

For magnetic label detection, we define a magnetic biosensor as a compact analytical device incorporating a biological/biologically-derived material associated with a physicochemical magnetic transducer [3]. Magnetic biosensors can be divided in detection methods employing magnetic labels [6,9] and label-free detection techniques [10,11]. The idea to employ a magnetic field as a transducer for detection of the large number of molecular recognition events at a time [1] stimulated intensive research in the field of magnetic label detection. A magnetic label detector is a device that measures variations of a magnetic field, converting them into a change of the light intensity, frequency, current, voltage, etc. [12,13].

For a long time, the development of magnetic biosensors for magnetic label detection was suppressed by the absence of large batches of MNPs playing roles of magnetic labels [13]. The large size of the batch is a crucial request of nanomedicine [14,15,16]. Applications of magnetic nanoparticles in chemistry, biology, and biomedicine are not limited by the magnetic biosensors. The large surface-to-volume ratio greatly affects the physical and chemical properties of MNPs and provides their high adhesion activity. The size and shape of the nanoparticles can be controlled by the modification of the conditions of their synthesis [17,18]. The MNPs provide an advantageous platform for the immobilization of biomolecules. Their efficient interaction with biomolecules and cells, together with their high penetrating ability, allows using them for development of biosensors, cell imaging, cell labeling, and cell manipulation [19,20]. The size of the batch is important, but it is not the only parameter crucial for the success of the biomedical applications. Other parameters are the average size of the particles, their shape, particle size distribution, etc. Concern regarding the width of particle size distribution, in some sense, is a matter of choice. There might be applications that demand unimodal PSDs, but there might also applications for the ensemble of MNPs with a certain size distribution. One such concern is related to the approach proposed in the work of [14]. MNP sizes, in the case of angiogenesis treatments, can be rather large, as the pore sizes in the blood vessels of the tumor are larger and show variety of the sizes and shapes, in comparison with the geometries of the normal blood vessels.

Carefully tuned magnetic properties of MNPs significantly increase the sensitivity of the assays of MNP-based biosensors [21] and allow for the specific magnetic separation of nanoparticle-labeled biocomponents. Anyway, the application of the gradient’s external magnetic field provides a tool for the controlled and directional movement of nanoparticles and nanoparticles-labeled objects [3,22], thus providing the opportunity for using them in medicine via magnetic resonance imaging (MRI) scanning for targeted drug delivery and magnetic hyperthermia [16,23,24,25].

The materials attracting the highest interest, due to their high biocompatibility, are the following iron oxides: γ-Fe_2_O_3_ (maghemite) and Fe_3_O_4_ (magnetite) [26]. Among others, iron oxide MNPs synthesized by the laser target evaporation (LTE) have some advantages for biomedical applications [4,19,27,28]. LTE is a method of high-energy pulse evaporation of an oxide with consequent gas-phase condensation of spherically-shaped MNPs. The method is highly productive, cost-efficient, and ecologically friendly. It provides large batches, of the order of 100 g of spherical iron oxide MNPs, with excellent batch-to-batch reproducibility [25,28,29].

The magnetic properties of the materials can, in the simplest way, be classified in three big groups: diamagnetic, paramagnetic, and ferromagnetic. In the majority of cases (except desoxygenized erythrocytes), cells and tissue have diamagnetic properties [30,31]. This means that MNPs, even in the superparamagnetic state [30,31], become highly “visible” or “countable” for the magnetic measurements [32,33]. Magnetic fields penetrate biological tissues and, therefore, can be exploited for diagnostic or/and therapeutic applications in combination with MNPs. Biological effects caused by the presence of magnetic nanoparticles of iron oxides were under special interest, including the cases of γ-Fe_2_O_3_ LTE maghemite, both without and in the presence of external magnetic field [34,35].

Both the detection of biomagnetic fields, such as magneto cardio-, encefalo-, or miograms (field strength below 10^−8^ Oe), and application of a rather strong external magnetic field (up to 50 kOe) are required by present-day biomedical applications [30]. In magnetic biosensors, depending on the particular type of the device, there are uniform, gradient, constant, or alternating magnetic fields. Gradient fields are used for creating magnetic forces above the values of the forces corresponding to gravity thermal fluctuations and liquid media viscosity. As an example, one can mention the need to remove non-immobilized magnetic labels during the functionalization process [1]. A typical value of the external constant magnetic field of magnetic biosensor, working on the principle of magnetoimpedance, is about 3–5 Oe for the work point, i.e., the middle of the interval of maximum sensitivity [3,19]. However, fields up to 100 Oe can be used for the technological operation of the device. This means that the operation of a magnetic biosensor, on one hand, includes the application of magnetic fields and, on the other hand, allows us to apply magnetic fields with certain parameters, if necessary. Therefore, understanding the contribution of the external magnetic field to the properties of biological objects with MNPs is an important direction of the research.

The application of MNPs in medicine requires an assessment of their biological activity. In particular, the possible impact of MNPs on the secretion of pro-inflammatory cytokines can cause a local, or even systemic, inflammatory reaction, leading to pulmonary edema, hemorrhage, vascular thrombosis with the multiple organ failure, shock, and death [36]. Thus, the biocompatibility of MNPs is a very important issue in the development of magnetic biosensors.

In this work, the biological effect of the γ-Fe_2_O_3_ magnetic nanoparticles obtained by laser target evaporation was investigated in vitro for the primary cultures of human dermal fibroblasts. The effects for the MNPs uptaken by cells and MNPs contained in culture medium were analyzed for the fibroblast’s proliferation and secretion of cytokines and collagen in the presence, or absence, of a constant external magnetic field created by a specially designed magnetic system.

## 2. Materials and Methods

### 2.1. Preparation of Magnetic Nanoparticles and Ferrofluid

Iron oxide MNPs were synthesized by laser target evaporation (LTE) method [4,19,28,29], using laboratory setup with Ytterbium (Yb) fiber laser with 1.07 µm wavelength. The target was pressed from the commercial microparticles of the magnetite (Alfa Aesar, Ward Hill, MA, USA) powder. The laser beam was focused onto the target surface by optical system Optoscand d25 f60/200, with 200 mm focal length. The diameter of the focal spot was as high as 0.45 mm. The cylindrical target was 65 mm in diameter and 20 mm in height. It was mounted in a holder, which provided rotation and horizontal displacement of the pellet. It resulted in 20 cm/s beam scan rate on the target surface, which ensured uniform wear-out of the target surface. The laser operated in a pulsed regime, with pulse frequency 4.85 kHz, pulse duration 60 μs, and output power 212 W. The working gas was a mixture of N_2_ and O_2_ in the volume ratio 0.79:0.21. It was blown into the evaporation chamber at a flow rate 58 L/min at normal pressure. The linear flow rate at the target surface was approximately 12 m/s. The oxide vapors were driven away from the focal spot and condensed in spherical nanoparticles, which were moved by the working gas into the cyclone and fine filter where the powder had been collected.

The stock suspension (ferrofluid) of air-dry MNPs in 5 mM sodium citrate was de-aggregated by ultrasound treatment for 30 min using Cole-Parmer CPX-750 processor (Cole-Parmer, Vernon Hills, IL, USA) operated at 250 W. Permanent cooling of the suspension was provided. The remained aggregates were eliminated by centrifuging at 8000 rpm for 5 min (Hermle Z383 centrifuge; Hermle AG, Gosheim, Germany) to provide stability of the ferrofluid in high ionic strength electrosteric stabilizer ammonium poly(methacrylate) brand mark Darvan CN (Vanderbilt Chemicals, Norwalk, CT, USA) in 0.4% (wt.) concentration. The stock ferrofluid was then equilibrated in thermostat at 90 °C for 60 min. The final concentration of iron oxide MNPs in the stock ferrofluid was 4.24% (wt.). It was added to the cell culture media in pre-calculated portions to provide the desired concentration of iron oxide MNPs.

### 2.2. Characterization of Magnetic Nanoparticltes

The X-ray diffraction characterization (XRD) was performed using D8 DISCOVER diffractometer (Bruker Corp., Billerica, MA, USA) operated at 40 kV at Cu-K*α* radiation (wavelength *λ* = 1.5418 Å) with a graphite monochromator and scintillation detector. Bruker software TOPAS-3, with Rietveld full-profile refinement, was used for the quantitative diffractogram analysis. The chemical composition of LTE MNPs was determined by the combination of the analysis of the lattice period provided by XRD and Red-Ox titration (Schott Titroline, SI Analytics, Mainz, Germany). Weighted portions of MNPs were dissolved in hydrochloric acid under argon, and these solutions were automatically titrated with 0.2 N potassium dichromate solution. The electrical potential of an immersed Pt electrode to a reference AgCl standard electrode was measured simultaneously. The equivalency point on a titration curve gave the content of Fe(II) cations in solution. Separate titration was performed to determine Fe^2+^ content and total Fe content in MNPs. In the latter case, all Fe ions in solution were completely reduced to Fe(II) by the in situ hydrogen produced by the dissolution of a piece of Al wire in hydrochloric acid. Transmission electron microscopy (TEM) images of MNPs were obtained using JEOL JEM2100 microscope (JEOL Corp., Tokyo, Japan) operated at 200 kV.

The hydrodynamic diameter and zeta potential of species in ferrofluid were measured using Brookhaven ZetaPlus analyzer (Brookhaven Instruments, Holtsville, NY, USA).

Magnetic properties of MNPs (magnetic M(H) hysteresis loops) were measured at room temperature using a vibrating sample magnetometer (Cryogenics Ltd. VSM, London, UK). In our previous studies, we provided more detailed investigation of the MNPs obtained in similar conditions, and, therefore, more details can be found elsewhere [19,28].

### 2.3. Design and Characterization of Magnetic Matrix

One of the strict requirements of the biological studies is the analysis of a large, statistically supported data set. However, commercial systems for application of external magnetic field adapted for cell culturing are still not available. In our previous study [35], the magnetic matrix, tested based on 24-well polystyrene plate for cell culturing, was designed and tested for the case of human dermal fibroblast cell proliferation. In that study, the geometry of the magnetic matrix was the same as the geometry of 24-well polystyrene plate (Techno Plastic Products, Trasadingen, Switzerland): height 18.70 mm, length 127.80 mm, and width 85.60 mm. Although a relatively uniform constant magnetic field of about 500 Oe was created by the designed matrix at a distance of 3 cm, the uniformity zone covered only 6 central wells of the 24-well plate, providing somehow limited statistical data.

**Figure 1:**
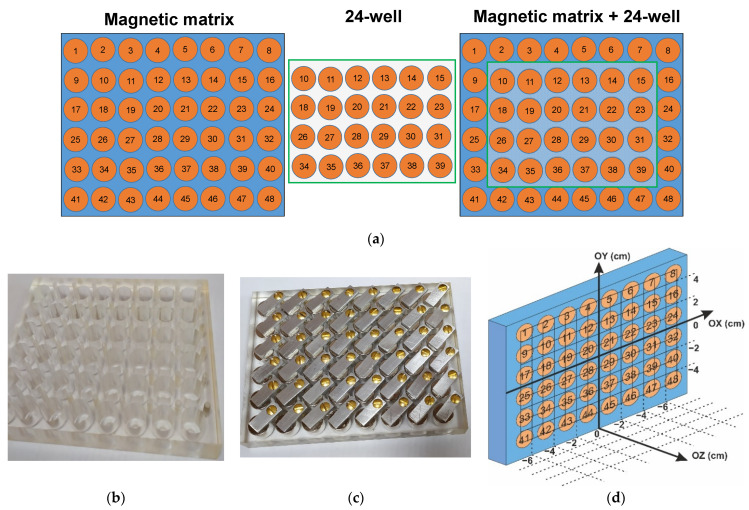
(**a**) Description of the magnetic matrix with 48 positions (left), 24-well plate the same geometry (centre), and 24-well plate on top of magnetic matrix (right); (**b**) polymer frame with selected 48 positions; (**c**) general view of magnetic matrix assembled with the permanent magnets; (**d**) coordinate system for the measurements of the magnetic field of the matrix.

Taking previous results into account, we designed a magnetic matrix with 48 positions of the same geometry of each well as for the 24-well standard plate, but at 8 × 6 positions in the of the weakly magnetic polymer frame (Figure 1a). The geometry of the polymer frame allows us to put 24-well plate on top of magnetic matrix, covering 24 positions in the center. Commercial cylindrical permanent NdFeB magnets of 15 mm in height and 14 mm in diameter were placed into each one of the 48 positions of the polymer frame (Figure 1b,c) and properly isolated, in order to avoid the magnets degradation. Thus, the matrix was divided into 48 zones of 4 cm^2^, each of which included one permanent magnet, and the poles all magnets were arranged accordingly.

**Figure 2:**
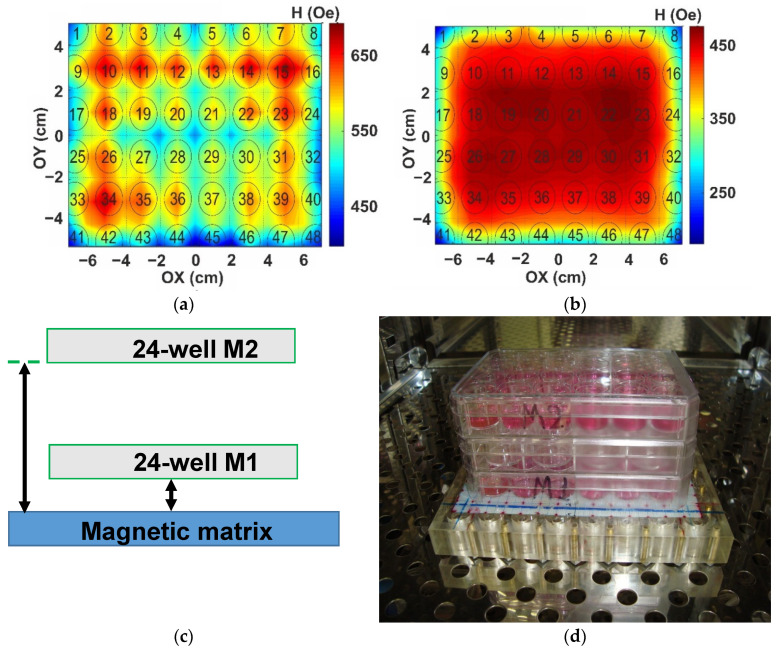
Constant magnetic field distribution of the external field created by the magnetic system in XY plane (see also Figure 1) at Z = 1.0 cm (**a**) and Z = 2.0 cm (**b**). Description of the distances M1 and M2 selected for cell experiments on the basis of magnetic measurements (**c**). Arrangement of the cellular experiments with the use of plates’ stack in the CO_2_ incubator (**d**).

Measurements of the magnetic fields created by the matrix were carried out using gaussmeter along three axes, with the zero position in the center of the plate (Figure 1d). The distances of 1.0, 2.0, and 5.0 cm along OZ axis were considered. Figure 2a,b shows experimentally obtained examples of the distribution of the constant magnetic field of the designed matrix at selected distances (being 1 and 2 cm, respectively). One can see that the central part of 24 wells corresponds to the area of the high uniformity of the external magnetic field. However, the average field strength value in this area depends on Z-coordinate.

The following values for the average magnetic field strenghts were obtained: H= 630 ± 30 Oe (for Z = 1 cm), H= 440 ± 20 Oe (for Z = 2 cm), and H= 320 ± 20 Oe (for Z = 5 cm). Figure 2a,b shows selected results of the matrix magnetic field measurements corresponding to the distances Z = 1.0 cm and Z = 2 cm from the surface of the magnets. One can conclude that designed new device ensures sufficiently uniform constant magnetic field in plane of the 24-well polystyrene plate for cell culturing for various distances. This means the increase of the statistical set of data from 6 (Ref. [35]) to 24 units in the present study. Finally, the distances Z = 1.0 cm (M1) and Z = 5.0 cm (M2) were selected because corresponding average values of the field strengths differed from each other by factor 2 for these positions. Figure 2c,d shows the definition of the distances M1 and M2 selected for cell experiments and photo of the experimental arrangement of the cellular experiments in the CO_2_ incubator, with the magnetic matrix at a bottom and two 24-well polystyrene plates for cell culturing placed at selected distances.

### 2.4. Design of Experiments with Cell Cultures

#### 2.4.1. Cell Culture

Human dermal fibroblasts were obtained from skin samples excised during surgery from four healthy donors. Prior to surgery, all donors provided the written informed agreement. The Ethics Committee of the Institute of Medical Cell Technologies (Ekaterinburg, Russia) approved the study.

Skin samples were minced into small pieces (below 1 mm^3^) and incubated in collagenase I solution (1000 U/mL) (Sigma-Aldrich, St. Louis, MO, USA) at 37 °C for 2.5 h. After incubation, the collagenase was neutralized by dilution with growth medium, and the tissue was dissociated into cell suspension by vigorous vortexing. After centrifugation, the supernatant was removed, and the cells were resuspended in growth medium and transferred to T25 culture flask (Nunc, Roskilde, Denmark). The growth medium consisted of the mixture Dulbecco modified Eagle’s medium (DMEM, Gibco, Thermo Fisher Scientific, Waltham, MA, USA) and medium F-12 (1:1) (PanEco, Moscow, Russia), supplemented with 12% of fetal calf serum (Biosera, Nuaille, France), glutamine (0.03%), and gentamicin (50 μg/mL). Cells were grown in CO_2_-incubator MCO-15AC (Sanyo/Panasonic, Moriguchi, Osaka, Japan) at 37 °C in an atmosphere with 5% CO_2_. Fibroblasts were passaged when they were 80–90% confluent, TrypLE (Gibco, Thermo Fisher Scientific, Waltham, MA, USA) was used for cell detachment. Prior to the experiments, all cell cultures were stored in liquid nitrogen. In the present study, the fibroblasts from passages 4–5 were used.

#### 2.4.2. Testing of MNPs Cytotoxicity

The experimental studies were conducted in two different ways: with short- and long-term incubation of fibroblasts with γ-Fe_2_O_3_ LTE magnetic nanoparticles. Short-term incubation was carried as follows. Cells were suspended in growth medium and seeded into the wells of 96-well cell culture treated plate (Eppendorf, Hamburg, Germany) at high density (6 × 10^4^ cell/cm^2^). Plates with cells were left overnight in CO_2_-incubator for cell adherence, then MNP suspensions in growth medium were added to plate wells. Cells with MNPs were incubated for 18 h in CO_2_-incubator; after incubation, the cell metabolic activity was measured by MTT test.

Long-term incubation was carried as follows. Cells were seeded in plate wells at the density of 2 × 10^4^ cell/cm^2^. Two hours after seeding, MNPs were added in plate wells. The metabolic activity of cells was measured after 120 h of incubation with MNPs in CO_2_-incubator by MTT test.

The MTT test is a colorimetric assay based on the reduction of a yellow tetrazolium salt (3-(4,5-dimethylthiazol-2-yl)-2,5-diphenyltetrazolium bromide). The growth medium was removed, and cells were washed from unbound MNPs and incubated with MTT (Acros Organics, Thermo Fisher Scientific, Inc., Waltham, MA, USA) solution (0.5 μg/mL, 100 μL per well) for 3 h at 37 °C. The formazan crystals were dissolved by 50 μL of dimethyl sulfoxide (DMSO) per well. The optical density was measured using microplate reader at 490 nm working wavelength, with 750 nm reference wavelength.

#### 2.4.3. Proliferation Assay

The proliferation assay was performed in 24-well cell culture plates in two conditions. Cell seeding was performed simultaneously for both conditions. The seeding density was as high as 5000 cells/cm^2^.

Condition I. Fibroblasts were grown in T25 culture flasks before seeding to plates. When the cell monolayer reached the 60–70% confluency, the MNPs were added in the final concentrations of 15, 4, or 2 µg/cm^2^. After 18 h incubation, cells were washed. The unbound MNPs were detached, and cells were suspended in growth medium free of MNPs. The suspensions were dispensed into the plates of the well.

Condition II: Fibroblasts were grown in the same way as in the case I in T25 flasks but without MNPs addition. After detachment, the fibroblasts suspension (without MNPs) was dispensed onto the plates of the well. After 2 h, the MNPs were added in the final concentrations of 15, 4, and 2 µg/cm^2^.

Plates with cells (in both experimental conditions) were placed onto the magnetic matrix at a distance of 10 mm (M1) or 50 mm (M2), between the surface of matrix and cell monolayer. The control plates (no magnetic field applied) were placed outside the magnetic field. Both cells exposed to magnetic field and placed outside the magnetic field were incubated in CO_2_-incubator for 120 h, without medium change. After incubation samples of growth medium for ELISA (enzyme-linked immunosorbent assay) were collected, and the cells were fixed for cell counting.

#### 2.4.4. Cell Counting

Cells were washed 3 times with PBS, fixed by 2.5% glutaric aldehyde, and dehydrated by ethanol. Cell nuclei were stained by 300 nM 4′, 6-diamidino-2-phenylindole, dihydrochloride (DAPI) solution (Acros Organics, Thermo Fisher Scientific, Inc., Waltham, MA, USA). Cells with stained nuclei were photographed by fluorescent microscope Axio Lab A1 FL (Carl Zeiss, Oberkochen, Germany) in DAPI channel. Nine shots were taken for every well. Nuclei were counted using the ImageJ software (Wayne Rasband, NIH, Bethesda, MD, USA).

#### 2.4.5. Enzyme-Linked Immunosorbent Assay (ELISA)

The concentrations of interleukin 6 (IL-6), interleukin-8 (IL-8/CXCL8), and pro-collagen Iα were measured using Interleukin-6-ELISA-BEST (A8768, Vector-Best, Novosibirsk, Russia), Interleukin-8-ELISA-BEST (A8762, Vector-Best, Russia), and Human Pro-Collagen I alpha 1 DuoSet ELISA (DY6220-05, R&D Systems, Minneapolis, MN, USA) kits, respectively. The assays were carefully performed, according to the manufacturer’s protocols.

#### 2.4.6. Prussian Blue Staining

After fixing cells with glutaric aldehyde and ethanol, the monolayer was covered with the staining solution containing 2% of hydrochloric acid and 2% of potassium ferrocyanide (K_4_[Fe(CN)_6_]). After 15 min, the staining solution was removed, and the cell monolayer was washed 3 times with distilled water.

#### 2.4.7. Colorimetric Test for Collagen Deposition Measuring

The quantity of collagen deposited in the plates was measured after nuclei photographing. The measurement was achieved by staining with Sirius Red using Sirius Red/Fast Green collagen staining kit (#9046, Chondrex, Woodinville, WA, USA), according to the manufacturer’s protocol.

#### 2.4.8. Statistics

All experiments were conducted in four independent repeats. Employed fibroblast cultures were obtained from different donors. Three-four technical replicates were performed for each repeat. The cell monolayer densities, optical densities, and concentrations of secreted products were normalized to the corresponding values obtained for controls. The use of relative values was necessary, in order to avoid the effect of the individual features of the fibroblast cultures from different donors on the results of experiments.

Statistical data processing was performed using the application software package “STATISTICA 6.0” (Statsoft, Dell, Round Rock, TX, USA). Statistically significant differences between groups in pairwise comparison were assessed using the one-way ANOVA test. For comparison of multiple groups with a control group the Dunnett’s testing was performed. The Kolmogorov–Smirnov test was used for testing the distribution normality. The dependences between concentrations of secretory products and MNPs presence were evaluated using Spearman’s rank correlation coefficient. The statistical significance was accepted for *p* < 0.05.

## 3. Results

### 3.1. Properties of γ-Fe_2_O_3_ LTE Magnetic Nanoparticles and Ferrofluid

**Figure 3:**
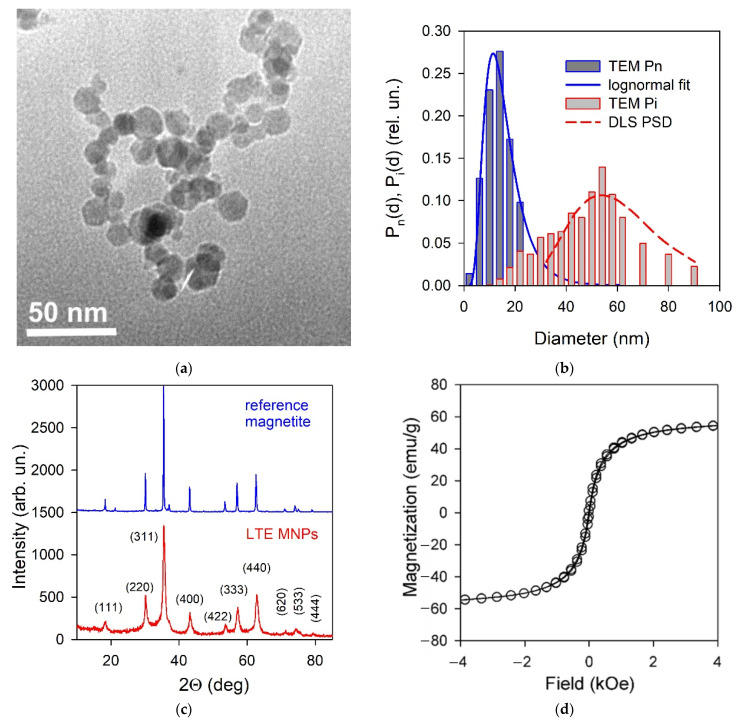
(**a**) Transmission electron microscopy (TEM) image of iron oxide MNPs. (**b**) Particle size distributions, based on the graphical analysis of 2572 TEM images. Histogram filled in dark grey corresponds to the numerical fraction of particles in a certain range of diameters. Blue line is the lognormal fitting of the histogram. Histogram filled in light grey corresponds to the intensity average distribution calculated from TEM images. Dashed red curve is the intensity average PSD measured via dynamic light scattering. (**c**) XRD pattern of iron oxide MNPs with Miller indexes, in comparison with the reference XRD pattern for magnetite (Alfa Aesar, Ward Hill, USA). To avoid overlay, the reference pattern is shifted upward by 1500 arb. Un. (**d**) Hysteresis loop of iron oxide MNPs at 300 K.

Figure 3a shows typical TEM image of iron oxide MNPs. The shape of MNPs was close to being spherical. Although rare, polyhedral distortions in the shape of several particles might also be marked out. The graphical analysis of 2572 TEM images of the individual particles provided a histogram of particle size distribution by their number fraction, which is provided by a histogram filled in dark grey in Figure 3b. The blue line in the figure corresponds to the lognormal fitting of the particle size distribution (PSD). According to it, the mean particle diameter in the ensemble was 14.1 nm, and the geometric standard deviation of the distribution was 1.585.

Figure 3c shows the XRD pattern of iron oxide MNPs with Miller indexes. The diffractogram corresponded to the inverse spinel crystalline structure of Fd-3m space group, which was typical for magnetite—the XRD pattern is presented in Figure 3c, as a reference. Meanwhile, some peaks slightly deviated from their positions. Hence, the composition of iron oxide MNPs did not exactly fit Fe_3_O_4_. It is related to the distortions of the magnetite lattice, owing to the deficiency in the iron sub-lattice [28]. The refinement of the chemical composition of LTE iron oxide MNPs was performed via Red-Ox titration, using potassium dichromate as an oxidizing agent. The analysis was performed for MNPs dissolved in the excess of hydrochloric acid. This solution contained both Fe^3+^ and Fe^2+^ ions, in the same ratio as in MNPs. Figure S1, in supporting information, provides the titration curve for the solution of a mixture Fe^3+^/Fe^2+^, as it had been received directly, and the titration curve for the solution with all Fe ions totally reduced to Fe^2+^. The shift between these two curves depended on the Fe^3+^/Fe^2+^ ratio in the MNPs composition. The value of the Fe^3+^/Fe^2+^ ratio, calculated from equivalency points on titration curves, was found equal to 34. In the magnetite (Fe_3_O_4_) composition, it equals 2; in Fe_2_O_3_ composition, it is infinite. This means that the composition of LTE MNPs was close to maghemite—γ-Fe_2_O_3_. The prefix “γ” marks out that the crystalline structure is still that of magnetite but not hematite. In more detail, the crystalline structure of magnetite/maghemite is provided elsewhere [28].

Figure 3d shows the magnetic hysteresis loop of iron oxide MNPs at 300 K. It has a typical shape for the magnetically soft particles with very low coercivity (H_c_ = 30 Oe) and remanence. The value of saturation magnetization (M_s_) at 1.8 kOe was close to 60.5 emu/g, i.e., it is about 25% below the Ms value for a bulk maghemite. Even so, it was close to the typical values for maghemite MNPs of the size under consideration, as obtained by LTE and other techniques and reported in the literature [27,31,32]. For the maximum field used in the present work for M(H) loops measurements, it was consistent with the core-shell model (based on the random anisotropy model) describing the magnetic structure of the particle as a ferrimagnetic core and surface shell, where the spins were frozen with no long-range magnetic order.

The characterization of the state of the aggregation of iron oxide MNPs in stock ferrofluid was achieved by means of dynamic and electrophoretic light scattering. The PSD in the stock suspension, obtained via DLS in the unimodal regime, is provided in Figure 3b as a red dashed curve. According to basic principles of the method, it is an intensity-based distribution *P_i_*(*d*), which means that the ordinate provides the fraction of the intensity of the scattered light provided by MNPs in the corresponding range of diameters. It is different from the numerical distribution *P_n_*(*d*) provided by a blue line in Figure 3b, which is the number fraction of MNPs in a certain range of diameters. Meanwhile, given the actual numbers of MNPs in the ensemble, the intensity-based distribution *P_i_*(*d*) can be calculated using an equation:

$$P_{i}(d_{j})=\frac{d_{j}^{6}N_{j}}{\sum d_{j}^{5}N_{j}},$$

where *d_j_* is the average diameter of particles of the *j*-th fraction, and *N_j_* is the number of particles in this fraction. The calculated intensity-based PSD is provided in Figure 3b via the histogram filled in dark grey. It is noticeable that the PSD measured via DLS is in good agreement with the histogram calculated based on TEM images for individual particles. This means that the stock suspension contained individual MNPs, rather than their aggregates. Such a de-aggregation of air-dry MNPs in suspension is, in general, thermodynanically unfavorable and can be provided only with the use of stabilizers. In the present study, we used sodium citrate as an electrostatic stabilizer. Citrate anions were adsorbed at the surface of MNPs and provide net negative charge of MNPs, which prevented their aggregation, due to the electrostatic repulsion. The zeta potential of the ferrofluid was determined at –60 mV via electrophoretic light scattering. The value provided the basis for the high colloidal stability of the ferrofluid. Due to this, the stock suspension did not sediment in at least a one-month period. However, its long-term stability, and the stability under the variation of different internal and external factors, is yet unclear and to be established in further separate studies.

### 3.2. Biological Activity of MNPs

#### 3.2.1. Cellular Uptake and the Cytotoxic Effect of γ-Fe_2_O_3_ LTE MNPs on Fibroblasts

**Figure 4:**
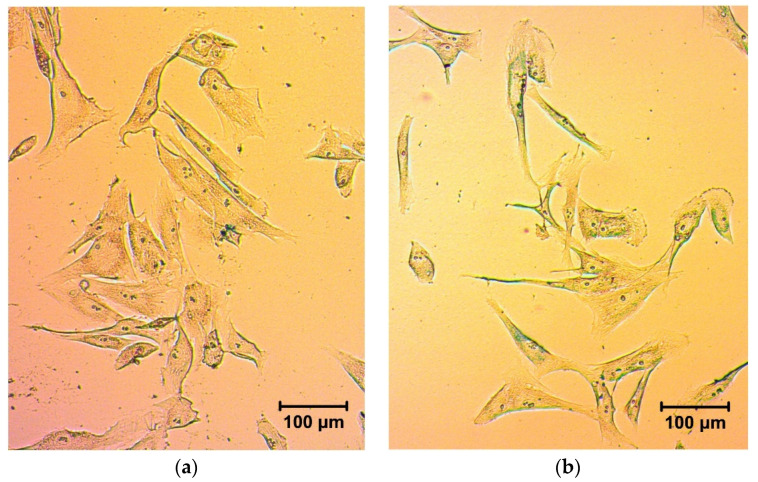

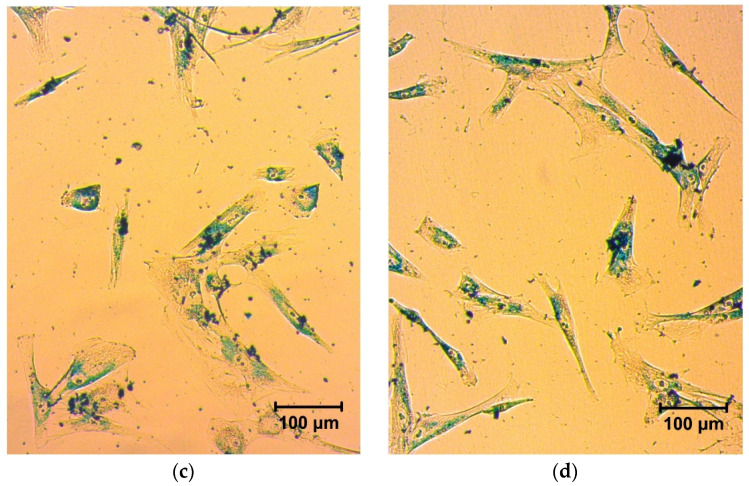
Prussian blue staining of skin fibroblasts after incubation with MNPs. Cells were incubated for 18 h with MNPs in various concentrations, washed from unbound nanoparticles, detached, seeded on culture-treated plastic, incubated for 18 h, fixed, and stained. (**a**) control without MNPs; (**b**) 2 μg/cm^2^; (**c**) 32 μg/cm^2^; (**d**) 512 μg/cm^2^. Magnification × 100.

In the first stage of the biological experiments, the MNPs uptake by fibroblasts was checked. The MNPs were added to cell monolayers in various concentrations. After 18 h, the cells were fixed and stained by Prussian blue. The observed blue staining of cells was the most intensive at high concentrations of MNPs. Anyway, when fibroblasts with MNPs were detached by TrypLE, washed, and seeded again on the plastic surface, they were still stained by Prussian blue, which confirms the internalization, or, at least, strong binding of γ-Fe_2_O_3_ LTE MNPs by the fibroblasts (Figure 4).

**Figure 5:**
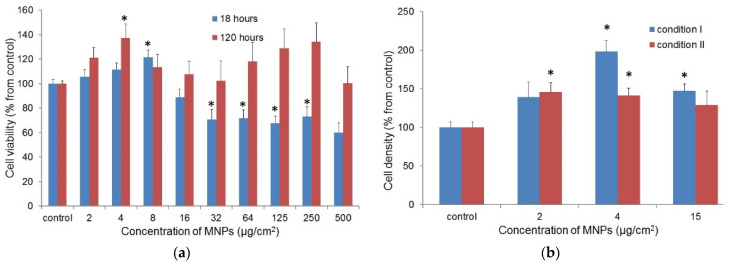
(**a**) The metabolic activity of human skin fibroblasts after short- (blue columns) and long-term (red columns) incubation with MNPs in various concentrations (MTT test). The data presented are normalized to optical density (OD) in control without MNPs (accepted for 100%). Values that are statistically different from control (*p* < 0.05) are indicated by asterisks. (**b**) Proliferation of fibroblasts in the presence of MNPs. The cells were seeded with uptaken nanoparticles (condition I) or MNPs were added to growth medium after fibroblasts seeding (condition II). The data presented are normalized to cell monolayer density (cells/cm^2^) in control without MNPs (accepted for 100%). Values that are statistically significant from control (*p* < 0.05) are indicated by asterisks.

The effect of MNPs on the metabolic activity of fibroblasts was measured by MTT test. The assay was performed in two conditions to evaluate the short-term effect of MNPs (18 h) and their long-term effect (120 h) on proliferating cells. Figure 5a shows the obtained results.

The MNPs did not cause the complete loss of viable fibroblasts, even at the highest concentrations used in experiment. The reduction of metabolic activity in the experiment with short-term cultivation with LTE MNPs was more significant, in comparison to the condition of long-term cultivation. After short-term incubation with nanoparticles, the partial loss of metabolic activity of fibroblasts was observed; at long-term cultivation, the cytotoxic effect of MNPs on fibroblasts was not statistically significant (Figure 5a). At the same time, at certain low concentrations of MNPs, the metabolic activity of fibroblasts was significantly higher than in the control (8 μg/cm^2^ for short-term and 4 μg/cm^2^ for long-term cultivation, *p* < 0.05). It was expected that the higher metabolic activity of fibroblasts could be the result of their induced proliferation.

#### 3.2.2. The Effect of MNPs on the Proliferation of Fibroblasts

The contribution of the presence of MNPs to the proliferation and secretory activity of fibroblasts was evaluated for low MNPs concentrations of 15, 4, and 2 μg/cm^2^, using the results of the MTT assay. The effect of the MNPs was studied in two experimental conditions: cells with uptaken nanoparticles in growth medium free of MNPs (I) and cells in growth medium with MNPs (II). Fibroblasts seeded in both conditions were grown for 120 h. Figure 5b shows the obtained results.

The presence of MNPs caused an increase of the fibroblasts monolayer density for both experimental conditions. In most cases, the difference from the control was statistically significant (*p* < 0.05). The highest fibroblasts density was observed at the MNPs concentration of 4 μg/cm^2^, when cells with uptaken nanoparticles were seeded (condition I).

#### 3.2.3. Cytokine Release

**Figure 6:**
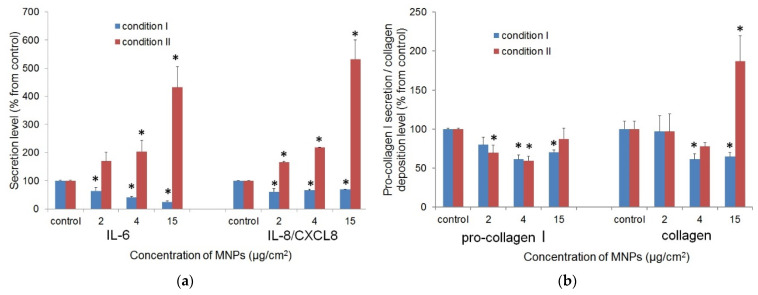
(**a**) Effect of MNPs on fibroblasts IL-6 and IL-8 secretion. Growth medium after fibroblasts incubation with MNPs was used for ELISA. The data presented were normalized to secretion level (pg of secreted product per 1000 cells) in control without MNPs (accepted for 100%). Values that are statistically different from control (*p* < 0.05) are indicated by asterisks. (**b**) Pro-collagen I secretion and collagen deposition by fibroblasts. The quantity of pro-collagen I released and collagen deposited (ng per 1000 cells) in MNP-free control was accepted for 100%. Values that are statistically different from control (*p* < 0.05) are indicated by asterisks.

The impact of LTE MNPs and constant magnetic field on cytokine production for fibroblasts was estimated using the ELISA methodology. The concentration of released cytokines was measured in their quantity per 1000 cells. The relative IL-6 and IL-8 secretion levels are shown in Figure 6a.

The fibroblasts grown in the medium with LTE MNPs (condition II) demonstrated increased levels of IL-6 and IL-8 secretion. In the most cases, the differences from the control values were statistically significant (*p* < 0.05). The impact of MNPs in growth medium on IL-6 and IL-8 secretion was further confirmed by the positive correlation between the nanoparticles concentration and cytokines secretion level (*p* < 0.05), regardless of presence/absence of the constant magnetic field. However, the uptaken LTE MNPs in fibroblasts did not stimulate cytokine secretion. Instead, in most cases, there was a statistically significant decrease of cytokines production.

#### 3.2.4. Collagen Secretion and Deposition

The collagen secretion was estimated by two parameters: the quantity of pro-collagen I in growth medium (ELISA) and quantity of collagen deposited by cells (Sirius Red staining) per 1000 cells. The MNPs in the growth medium (condition II) and uptaken by fibroblasts (condition I) did not cause the significant increase of pro-collagen I production; the secretory activity of fibroblasts was, rather, impaired (Figure 6b). The estimation of the quantity of deposited collagen confirmed this result. The unexpectedly high calculated values for collagen deposition at the MNPs concentrations of 15 μg/cm^2^ (condition II) seemed to be the result of non-specific binding of Sirius Red by the MNPs (Figure 6b).

#### 3.2.5. Effect of Magnetic Field on the Proliferation and Secretory Activity of Fibroblasts

The effect of the constant magnetic field on fibroblasts proliferation and secretion was measured at field strengths of 630 ± 30 and 320 ± 20 Oe. Measurements were performed using MNPs in the concentrations mentioned earlier (no MNPs and 2, 4, and 15 μg/cm^2^) in both experimental conditions. For each concentration of LTE MNPs, the obtained data were normalized to the control (cells without exposure to the constant magnetic field).

**Figure 7:**
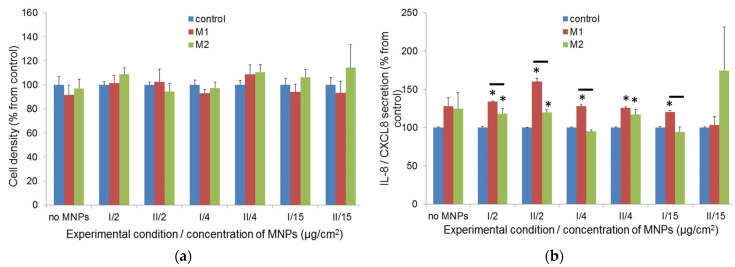
(**a**) Fibroblasts proliferation activity with application of the constant magnetic field at various concentrations of MNPs. Cells were seeded with uptaken nanoparticles (condition I), or MNPs were added to growth medium after fibroblasts seeding (condition II). Fibroblasts were incubated outside magnet or exposed to M1 and M2 magnetic field. For every MNPs concentration, the data were normalized to the cell monolayer density in the absence of magnetic field (accepted for 100%). No statistically significant differences in proliferation between cells exposed to constant magnetic field and control were found at all MNPs concentrations. (**b**) IL-8/CXCL8 secretion by fibroblasts with application of the constant magnetic field. For every MNPs concentration, the data are normalized to secretion level in the absence of magnetic field (accepted for 100%). Values that are statistically different from control (*p* < 0.05) are indicated by asterisks; significant differences between M1 and M2 are indicated by lines.

No effect of a constant magnetic field on the fibroblasts proliferation was observed, regardless the presence or absence of LTE MNPs (Figure 7a). At all concentrations of MNPs, and for both conditions, there were no statistically significant differences between the monolayer densities of fibroblasts exposed to the constant magnetic field and without such an exposure (*p* > 0.05).

The magnetic field did not cause significant deviations in IL-6 production (data not shown). At the same time, the IL-8 secretion level in the constant magnetic field was higher than in the control without field application, regardless of the method of adding MNPs. These differences were mostly statistically significant (Figure 7b).

Exposure to a magnetic field did not result in significant variations in collagen producing in most instances. Statistically significant differences (*p* < 0.05) were found in two cases each for pro-collagen I secretion (15 μg/cm^2^, condition I: M1 > M2; 2 μg/cm^2^, condition I: M1 > M2) and collagen deposition (control without MNPs: M1 > M2; 4 μg/cm^2^, condition II: M1 > M2).

## 4. Discussion

In recent years, the request for the development of new types of magnetic compact analytical devices (including MNPs based applications) has steadily grown [37]. Development of electrophysical techniques made large quantities of MNPs available, as obtained in the single batch fueling of the development of nanopharmaceutical drugs [14,15,29]. LTE MNPs were used for hyperthermia, regenerative medicine, and magnetic ferrogel detection via magnetic field sensors [6,27]. The last detection case supported the next step from the single cell to the natural tissue state evaluation [38]. However, the biological activity of MNPs, using the primary cultures of human skin fibroblasts, are still underdeveloped. Fibroblasts are widely distributed in connective tissue and responsible for the secretion of growth factors, cytokines, and components of extracellular matrix (ECM). These cells actively participate in wound healing, as well as the regulation of inflammation reactions. They also play role in pathogen recognition and the initiation of the immune response [39].

The results of MTT tests (see Figure 5a) demonstrated the low cytotoxicity of MNPs for fibroblasts, even at high concentrations, which is consistent with other research [40]. Interestingly, the cytotoxic effect of MNPs at long-term incubation was less pronounced than at short-term. The metabolic activity of fibroblasts after 5-days incubation with nanoparticles in high concentrations did not differ significantly from the control. It was suggested that the cytotoxic effect of MNPs is mostly due to some primary injury of cell membrane by precipitating nanoparticles [41]. At the same time, the presence of MNPs at low concentrations even stimulated the metabolic activity of fibroblasts (Figure 5a), which could reflect the enhanced proliferation of the cells.

In the next series of experiments, the possible effect of LTE MNPs in low concentrations on the proliferation of fibroblasts was evaluated by direct cell counting. The experiment was performed in two conditions to distinguish the effect of MNPs after uptake by cells (I) and MNPs in growth medium (II). The concentrations of the MNPs selected for experiments were based on the results of MTT tests. It was demonstrated that MNPs in low concentrations enhanced the proliferation of fibroblasts in both conditions (see Figure 5b). This result demonstrates that the presence of MNPs uptaken by cells was sufficient for the stimulation of cell proliferation, regardless of presence MNPs “outside” cells in growth medium.

The stimulation of cell proliferation by MNPs is a rather interesting phenomenon, since most of research reports the deleterious effect [42,43] or absence of effect of magnetic nanoparticles on cell viability/proliferation [44]. There are some works describing the positive impact of MNPs on mesenchymal stem cells (MSCs) proliferation [45]. The cytotoxicity of MNPs is usually explained by the increased production of ROS (reactive oxygen species) in cells [43]. Even so, the mechanism of the stimulatory effect of MNPs on cell proliferation remains unclear. It is not excluded that the uptaken MNPs are just the extra sources of ferric ions for cell metabolism.

The results of ELISA (see Figure 6a) demonstrated that the incubation of MNPs with fibroblasts boosted cytokine secretion. The positive correlation between the quantities of released cytokines and MNPs concentration in growth medium supports the stimulatory effect of nanoparticles on IL-6 and IL-8 production. At the same time, the fibroblasts with uptaken nanoparticles (condition I) did not demonstrate enhanced cytokine secretion. We can conclude that MNPs-induced cytokine secretion is caused by interaction of nanoparticles with the cell surface.

The synthesis of IL-6 and IL-8 is activated via toll-like receptors (TLRs)-dependent signaling pathways [46,47]. All types of TLRs (TLR1-TLR10) are expressed in fibroblasts [48]. We can assume that MNPs non-specifically bind TLRs and activate intracellular signaling cascades that initiate the excessive cytokine production. The alternative possible explanation: the injury of cell membrane by MNPs cause the release of danger-associated molecular patterns (DAMPs). DAMPs can also stimulate cytokines secretion via interaction with TLRs and other pathogen-recognition receptors.

The impact of MNPs on growth factor secretion has been notified by other research [44]. They have shown that MNPs stimulate MSCs for the vascular endothelial growth factor and some other cytokine secretions. It was suggested that the enhanced MSCs expansion in the presence of MNPs was induced by the modulation of the cytokine production of cells [45]. Meanwhile, this explanation is not consistent with the results of our research: MNPs uptaken by fibroblasts (condition I) stimulated the cell proliferation, albeit the cytokine secretion level was not elevated.

At the same time, MNPs did not demonstrate the stimulatory effect on pro-collagen secretion (see Figure 6b). Conversely, the quantity of pro-collagen (per 1000 cells), secreted by fibroblasts with MNPs, in most cases, was significantly lower, than in control group. It seems that this depletion of collagen secretion is not related to MNPs activity but, rather, due to the higher fibroblasts density in the samples with nanoparticles. The lower collagen secretion by fibroblasts in the monolayer with higher cell density was mentioned in a number of studies [49].

The impact of weak constant magnetic field on the fibroblasts (see Figure 7) with MNPs and MNPs-free fibroblasts was tested in parallel with the use specially designed magnetic system. The results of the studies of the magnetic field effect from other researchers are controversial. In some works, the exposure of the cells to constant magnetic field reduced their proliferation rate [50,51,52]. Other research declared the acceleration of cell proliferation and even reduction of the toxic effect of MNPs [43,53] or absence of the effect of the applied field [54]. The data on the cytokines release are also variable. It was demonstrated that the exposure to a constant magnetic field, for both unlabeled adipose stem cells (ASCs) and ASCs labeled by iron oxide nanoparticles, moderately inhibits the expression of IGF-1, VEGF, and TGF-β1 mRNA [55]. The depletion of IL-6 production by myofibroblasts in a constant magnetic field, together with the absence of impact on IL-8 secretion, were also demonstrated [56]. In other studies, the enhancing effect of the constant magnetic field on VEGF secretion by mesenchymal cells was shown [57].

The mechanism of the interaction of a constant magnetic field and cell is not clear. It is often assumed that constant magnetic field affects the reactive oxygen species generation [58], while there are alternative hypotheses; for example, the constant magnetic field-induced activation of calcium channels in the cellular membrane, followed by influx of Ca^2+^ and cytoskeleton reorganization [59]. Obviously, the effect of constant magnetic field depends on the field strength, as well as the type and physiological features of the cells. The “normal” diploid cells are usually less sensitive to the field than cancer lines [34]. This agrees with our results, suggesting that exposure to weak static magnetic field did not affect the proliferation of fibroblasts, regardless of the presence of MNPs. The analysis of the secretion of IL-6 and pro-collagen/collagen did not show significant differences between the fibroblasts exposed to a constant magnetic field and those unexposed to it, except in few cases. Yet, the differences in these few cases looked occasional and did not show any tendency; hence, they did not demonstrate any effect of the external field application.

At the same time, some impact of the magnetic field on the IL-8 release has been noticed (see Figure 7b). The secretion level of this cytokine by fibroblasts exposed to a constant magnetic field was increased. Although the difference was not great (usually about 20–25%, compared to the control), this effect did not show the dependence on MNPs concentration, with respect to the way the MNPs were added. Hence, the constant magnetic field strength had a rather limited (if any) effect on fibroblasts proliferation and secretion. However, it is probable that strengthening the magnetic field would increase the effect on fibroblasts’ biological activity, making it more pronounced.

Even so, the fact that a constant magnetic field, of the order of several hundreds of Oersted, had no significant biological effects is very important for the development of the compact analytical devises. Earlier, we developed a magnetoimpedance sensor in the shape of meander, based on the [FeNi/Cu]4/FeNi/Cu/[FeNi/Cu]4/FeNi film element [60]. It was used for the detection of magnetic fields generated by the residues of γ-Fe_2_O_3_ ferrofluid, with very close characteristics to the studied in the present work. The quantitatively defined variation of the shape of magnetoimpedance curves after the application of ferrofluid particles on the surface of the elements was observed, opening a way for creating biomagnetic detection systems. The maximum sensitivity of the meander element was observed in the external constant magnetic field of about 8 Oe. Coming back to Figure 3d, one can estimate the magnetic moment corresponding to the operational field of 8 Oe for the MNPs under consideration, at about 1 emu/g. This is reasonably high, even for such a small field for designing the drug delivery system that is assisted by the magnetic field application and controlled by the magnetic field sensitive element but did not cause the biological effects.

In addition, over the last few years, we evaluated the biological effects of γ-Fe_2_O_3_ LTE magnetic nanoparticles for different biological samples, such as human mesenchymal stem cells, human leucocytes [4,61], Exophiala nigrum, Candida albicans, Staphylococcus aureus, and Escherichia coli [62,63]. The next step may include experimenting with the designed and developed magnetic matrix for the evaluation of the magnetic field biological effects.

## 5. Conclusions

γ-Fe_2_O_3_ magnetic nanoparticles with the mean diameter of 14.1 nm in the ensemble were obtained by the laser target evaporation electrophysical technique. Water-based ferrofluid, with a final concentration of MNPs with an iron oxide of 4.24% (wt.), was prepared for biological tests. The biological effects of the presence of MNPs were investigated “in vitro” for the primary cultures of human dermal fibroblasts. The effects of the MNPs contained in the culture medium or MNPs already uptaken by cells were evaluated for proliferation, and the secretion of cytokines and collagen. The MNPs demonstrated the moderate cytotoxicity of the fibroblasts. At long-term cultivation, the cytotoxic effect of MNPs was less pronounced than after short-term cultivation with fibroblasts. At low concentrations, the MNPs facilitate fibroblast proliferation. The stimulating effect was most likely caused by MNPs uptaken by cells, regardless of their presence in the growth medium. Anyway, the MNPs in the growth medium (but not the MNPs uptaken by cells) enhanced the release of pro-inflammatory cytokines (IL-6 and IL-8/CXCL8) via fibroblasts in a concentration-dependent manner. MNPs could activate the intracellular signaling of cytokines synthesis, either via nonspecific binding to TLRs or injuring cells and causing the release of DAMPs, as the MNPs did not cause the elevation of pro-collagen secretion and collagen deposition.

For the tests related to the role of the constant magnetic field in the biological activity of MNPs, the magnetic field system (having no commercial analogues), adapted to the size of standard 24-well cell culture plates, was designed, calibrated, and used. The exposure to the uniform constant magnetic field (H ≈ 630 or 320 Oe), oriented in the plane of the well, did not cause considerable changes in fibroblasts proliferation and secretion, regardless of the presence of MNPs. Statistically significant differences were detected only in the levels of IL-8/CXCL8 release. The field application did not significantly affect the cell proliferation, IL-6, and pro-collagen secretion, regardless of the presence of MNPs uptaken by cells or in growth medium. At the same time, the mild (though statistically significant) effect of the constant magnetic field was detected, with respect to IL-8/CXCL8 secretion. The obtained results can be useful for biomedical applications.

## Data Availability

Not applicable.

## Supplementary Materials

The following supporting information can be downloaded at: https://www.mdpi.com/article/10.3390/bios12080627/s1, Figure S1: Integral and differential plots of potentiometric titration using 0.2N potassium dichromate to determine chemical composition of LTE MNPs.
