# Supplementary material for: Biological Impact of γ-Fe2O3 Magnetic Nanoparticles Obtained by Laser Target Evaporation: Focus on Magnetic Biosensor Applications
Source: Biosensors (Basel). 2022 Aug 11;12(8):627. doi: 10.3390/bios12080627 (PMC9405828; doi:10.3390/bios12080627)
Supplement: Supplementary file 1 [file biosensors-12-00627-s001.zip › biosensors-1825053-supplementary.pdf]

Supplementary Materials

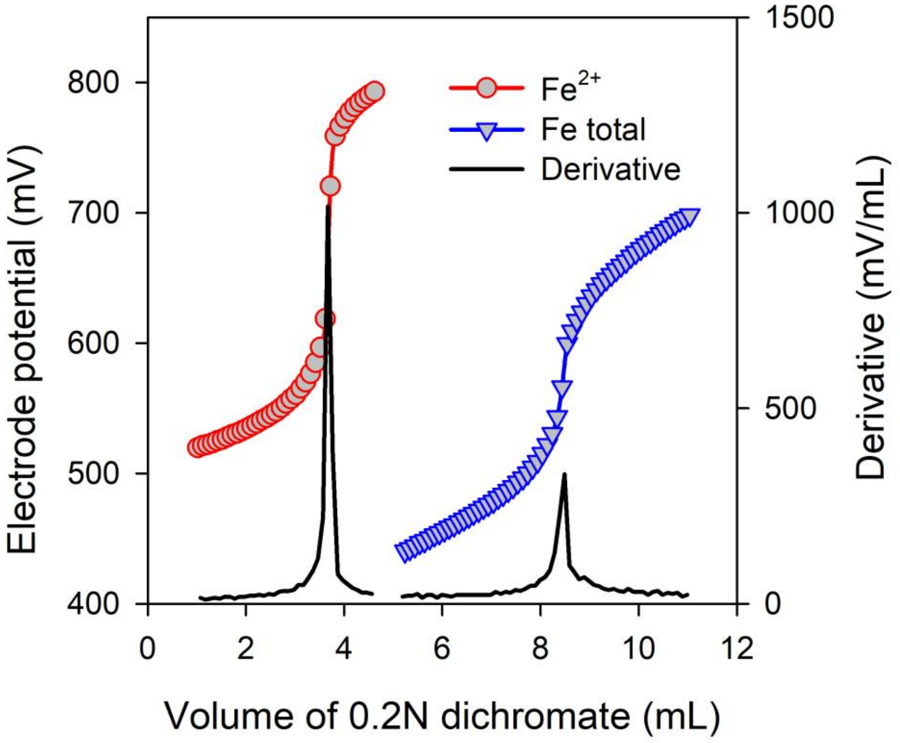

Figure S1. Integral and differential plots of potentiometric titration using 0.2N potassium dichromate to determine chemical composition of LTE MNPs. Red circles correspond to the titration of 300 mg of MNPs dissolved in hydrochloric acid. Blue triangles correspond to the titration of 100 mg of MNPs which first were dissolved in hydrochloric acid, and then all Fe ions were reduced to Fe^{2+} by the in situ hydrogen produced by the dissolution of a piece of Al wire.
